# Family Caregiver Burden of Medication Administration for Older Adults Admitted to Home Hospice

**DOI:** 10.1093/geroni/igab046.2934

**Published:** 2021-12-17

**Authors:** Jennifer Tjia, Margaret Clayton, Jennifer Smith, Olivia Wood, Susan Hurley, Geraldine Puerto, Vennesa Duodu, Susan DeSanto-Madeya

**Affiliations:** 1 UMass Chan Medical School, University of Massachusetts Medical School, Massachusetts, United States; 2 University of Utah, The University of Utah, Utah, United States; 3 Care Dimensions, Care Dimensions, Massachusetts, United States; 4 UMass Medical School, UMass Medical School, Massachusetts, United States; 5 University of Rhode Island, University of Rhode Island, Rhode Island, United States

## Abstract

OBJECTIVE To characterize FCG burden of medication administration for older adults in home hospice. METHODS Pilot clinical trial of a hospice-staff level communication and medication review program to facilitate goal-concordant prescribing, including deprescribing, for older adults in home hospice. Patients newly admitted to hospice were eligible if >=65 years, prescribed >= 5 medications and had a FCG. Exclusion criteria included being non-English speaking or having a Palliative Performance Score<40. Measurements include 24-item FCG Medication Administration Hassle Scale (range 0-96) at hospice admission and at 2-, 4-, 6-, 8-weeks and monthly until death. Descriptive statistics characterize baseline FCG Hassle score. RESULTS In this actively recruiting study, n=9 patient-caregiver dyads are enrolled to date. Mean patient age is 80.6 years (range 69-101). Of 9 caregivers, 7 were female, 5 children, and 3 spouses. The majority (67%) of caregivers were extremely involved in medication management. Mean FCG Hassle Score =17.1 (SE 5.9; range 2-58), and differed between spouses (mean =5 [SE 1.7; range 2-8]) and children (mean =31.4 [SE 9.53; range 3-58]). The highest burden concern was recognizing medication side effects, followed by feeling comfortable making medication decisions, arguing with the care-recipient about when to take medications, knowing why a medication is being given and whether it is effective, and knowing when to hold, increase, decrease a dose or discontinue the medication. CONCLUSION FCGs of older adults in home hospice report different levels of medication administration hassle depending on their relationship to the patient. The most bothersome concern is recognizing medication side effects.

